# The Self-Regulation of Eating Attitudes in Sport Scale: Defining an Optimal Regulation Zone

**DOI:** 10.3389/fpsyg.2022.905277

**Published:** 2022-07-21

**Authors:** Stéphanie Scoffier-Meriaux, Yvan Paquet

**Affiliations:** ^1^LAMHESS (UPR 6312), Université Côte d’Azur, Nice, France; ^2^Université de la Réunion, Saint-Denis, France

**Keywords:** regulation of eating attitudes, sport, zone of optimal regulation, eating disorders, scale

## Abstract

This study examines the hypothesis of a Zone of Optimal Regulation of Eating Attitudes in Sport (ZOREAS). The ZOREAS refers to a band, or zone within which athletes are most likely to optimize eating attitude regulation which should be associated with a low level of disordered eating. Scores outside this zone indicate a risk factor for eating disorders. One hundred and eleven volunteer athletes were recruited. Two variables were assessed: self-regulation of eating in sport, and eating attitudes and behaviors, measured with the French Self-Regulatory Eating Attitude in Sports Scale (SREASS), and the French version of the Eating Attitudes Test (EAT), respectively. Correlational analyses and an ANOVA were run. As expected, we observed a negative relationship between scores for the self-regulation of eating, and disordered eating attitudes in the sports context. To better-determine the ZOREAS, we ran a one-way ANOVA, which was significant for disordered eating attitudes. The plot of the interaction confirmed three trends: a high level of disordered eating attitudes (EAT scores over 20) is associated with a high level of self-regulation of eating attitudes (SREASS scores over 24); a medium level of disordered eating attitudes is associated with a low level of self-regulation of eating attitudes (SREASS scores under 19); a low level of disordered eating attitudes is associated with a medium level of self-regulation of eating attitudes (SREASS scores between 19 and 24). Thus, the ZOREAS was determined to be SREASS scores within the range 19–24. The ZOREAS may be useful to sports psychology practitioners who work with athletes.

## Introduction

Previous studies have defined behavioral self-regulation as the intentional control of attention, thoughts, emotions, behaviors, and the environment (Usher and Schunk, 2017; Baumeister et al., 2018). Self-regulation of eating attitudes is related to the individual’s concerns about his or her body, the intensity of exercise and physical activities (Desharnais et al., 1986; McAuley and Mihalko, 1998), and the degree of engagement in weight loss and nutrition programs (see Herman and Polivy, 2004, for a review). Behavioral self-regulation can be improved with practice (Hofmann et al., 2012) and the ability to effectively regulate one’s eating behavior is a major goal, not only for patients with an eating disorder, but also for individuals such as athletes. Although, in moderation, regulation is beneficial for the health of the athletes, the over-regulation of eating behavior can result in the person being underweight, or malnutrition. At the other extreme, a permanent failure to self-regulate eating may result in excess weight and obesity.

In the sport’s context, Scoffier et al. (2010a) developed and validated the French Self-Regulatory Eating Attitude in Sports Scale (SREASS). Five subscales measure the control of eating with respect to: (a) food temptation, (b) negative affect, (c) social interaction, (d) lack of compensatory strategies, and (e) a lack of anticipation of consequences on performance. The sport context is defined by specific socialization agents like the coach and specific norms of excellence and accomplishment (Scoffier et al., 2010a). In sport context, the athletes are characterized by subclinical pathology or at high risk for eating disorders and the SREASS is not specifically designed for individuals with eating disorders, like anorexia and bulimia. The SREASS give the particularly high stakes and intense social pressures of this context.

At the same time, Pine et al. (1997) reported a relevant correlation between symptoms of conduct disorders during adolescence and obesity in early adulthood, attributed to common mechanisms underlying “impulsive aggression” and dysregulation of body weight and this finding corroborated results reported in many of the studies cited in AbuSabha and Achterberg’s (1997) earlier review of the literature. For instance, the capacity for self-regulated eating was found to affect students’ control of fruit and vegetable consumption (Baranowski et al., 1997), and to be negatively related to disordered eating in the sports context (Scoffier et al., 2010a,b). In sport context the authors (Scoffier et al., 2010a,b) related the self-regulation of eating attitude scale to the eating attitude test (EAT)—26 in French version (Leichner et al., 1994). The EAT-26 is a generic scale to diagnose eating disorders however it is no specific to the sport context it is useful in the sport context. The instruments developed for daily living seem limited and understand the eating behavior through a validated tool for athletes seems needed to better understand the eating disorders in this population and to develop effective strategies for prevention.

In the general population, the self-regulation of eating has been directly related to positive physical and mental health outcomes, as well as overall life satisfaction (e.g., Sharbafshaaer, 2019; Gupta and Verma, 2020). On the other hand, low self-regulation is associated with a high body mass index and obesity (Ruzanska and Warschburger, 2019). Individuals who binge tend to overeat, while those with anorexia nervosa are likely to drastically reduce their food intake (Kenny et al., 2017, 2019). The ability to effectively regulate one’s eating behavior is a major goal, not only for patients with an eating disorder, but also for individuals engaged in dietary and weight-loss interventions (e.g., people who suffer from obesity). However, in previous articles higher eating self-regulation scores are a significant protective factor for a broad range of personal and interpersonal problems, and low self-regulation is a significant risk factor (e.g., Scoffier et al., 2010b). The contradiction between the higher eating self-regulation was observed in individuals with anorexia and the protective role of a higher eating self-regulation needs to be explored considering specifically the relationship between different level of self-regulation and disordered eating. Conversely, positive antecedents of disordered eating can eventually turn negative if taken too far. This can be characterized as the “too much of a good thing” effect (Pierce and Aguinis, 2013), which questions the unilateral goodness of self-regulation of eating attitudes. No research has investigated the curvilinear relationship between self-regulation of eating attitudes and disordered eating.

The literature defines two eating disorders, anorexia nervosa and bulimia nervosa, as the over- or under-control of socioemotional behaviors, respectively (Chen et al., 2015; Lynch et al., 2015). Disorders of over-control have been linked to social isolation, cognitive rigidity, highly-detail focused processing, a strong need for structure, and hyper-perfectionism (Lynch et al., 2015), traits that have also been found in individuals with orthorexia nervosa tendencies (Koven and Abry, 2015). It is possible that the latter individuals also suffer from poor emotional processing and regulation, and that orthorexic behaviors are used to regain control. Consequently, the aim of the present study was to identify optimal SREASS scores, a Zone of Optimal Regulation of Eating Attitudes in Sport (ZOREAS) and create categories which would be used by practitioners to identify athletes with attitudes associated with disordered eating. The categories would be based on the relationship between SREASS scores and EAT-26 scores. Scores outside the ZOREAS would indicate a risk factor for eating disorders.

## Materials and Methods

### Participants

*A priori* power analysis has been used to determine the necessary sample size *N* on G*Power (Faul et al., 2007). The ideal sample size *N* calculated is 66 participants. One hundred and eleven volunteer athletes (*M_age_* = 22; 51 years; *SD_age_* = 8.05) were recruited. The participants were at least 16 years old. They were 50 male athletes and 61 female athletes. They were regular sport practitioners, and all of them were at least regional competitors. They trained about 8 h per week on average, with 43.7% training more than 10 h per week and all of them training minimum 3 h per week. Three types of sport, considered as at-risk for disordered eating (Petrie and Greenleaf, 2007) were the focus of the investigation: aesthetic (*N* = 50), endurance (*N* = 36) and weight category (*N* = 25). The Research Ethics Committee of the University Côte d’Azur approved all procedures (authorization number: 2021-011).

### Background Information

At the beginning of the study, the following demographic data were collected: gender, age, sport type, and level of participation.

#### Self-Regulation of Eating Attitude in Sport

Self-regulation of eating attitudes in sports (Appendix 1) was assessed with the self-report SREASS questionnaire, developed, and validated in French by Scoffier et al. (2010a). This tool is composed of five factors pertaining to the self-regulation of eating attitudes in the following contexts: (a) food temptation, (b) negative affect, (c) social interaction, (d) lack of compensatory strategies, and (e) a lack of anticipation of consequences on performance. Items are measured on a Likert-type scale that ranges from “not at all able” (1) to “completely able” (6). A global index of self-regulation of eating attitudes was calculated by summing responses to items on all five subscales. The internal consistency of the global scale was satisfactory (*α* = 0.88).

#### The Eating Attitudes Test

Eating attitudes and behaviors were assessed with the French version of the Garner et al. (1982) Eating Attitudes Test (EAT, Appendix 2; Leichner et al., 1994). This 26-item self-report inventory comprises three subscales: dieting, bulimia, and oral control (e.g., “The desire to be thinner worries me”; “I cut up my food in small pieces”; “I vomit after eating”) and is used with both adolescents and adults. Participants respond to items on a six-point Likert-type scale ranging from 1 (always) to 6 (never). In the present study, and consistent with previous work (Scoffier et al., 2010a), only a global measure of disordered attitudes was used, and scores were reversed for the analyses. Thus, a higher score indicated a more disordered eating attitude. This global scale exhibited satisfactory internal consistency (*α* = 0.86).

### Procedure

The two questionnaires were administered online and could be completed in under 20 min. Data were collected *via* the Lime Survey interface of the Université Côte d’Azur. All data were anonymous; participants were informed beforehand that they were not obliged to respond to every question, that this was not a test (i.e., there were no right or wrong answers), and that all responses would remain strictly confidential and only used for research purposes.

### Analyses

Means, SDs, alpha coefficients, and bivariate correlations were calculated for the self-regulation of eating attitudes and the disordered eating variables. We adopted a procedure based on hierarchical linear regression analyses with self-regulation of eating attitudes on disordered eating, and a quadratic model (self-regulation of eating attitudes on disordered eating with the addition of a quadratic term) to determine the curvilinear relationship between the variables. An ANOVA was run to determine if there are self-regulation of eating attitudes’ mean differences between the groups. Follow-up analyses examined simple main effects. Data were analyzed using SPSS version 22.

## Results

Descriptive statistics are presented in Table 1. Twenty-five participants scored over 20 on the EAT scale and were considered as susceptible to developing an eating disorder. The correlation matrix (Pearson’s *r*; Table 1) highlighted a negative relationship between SREASS and EAT scores among the overall sample.

**Table 1:** Descriptive statistics and *post-hoc* tests.

	*N*	*M*	SD	*r*
SREASS	111	19.92	3.84	−0.53^*^
EAT	111	14.84	9.36	−

*r* is the correlation between SREASS and EAT scores.

* *p* < 0.05.

**Figure 1:**
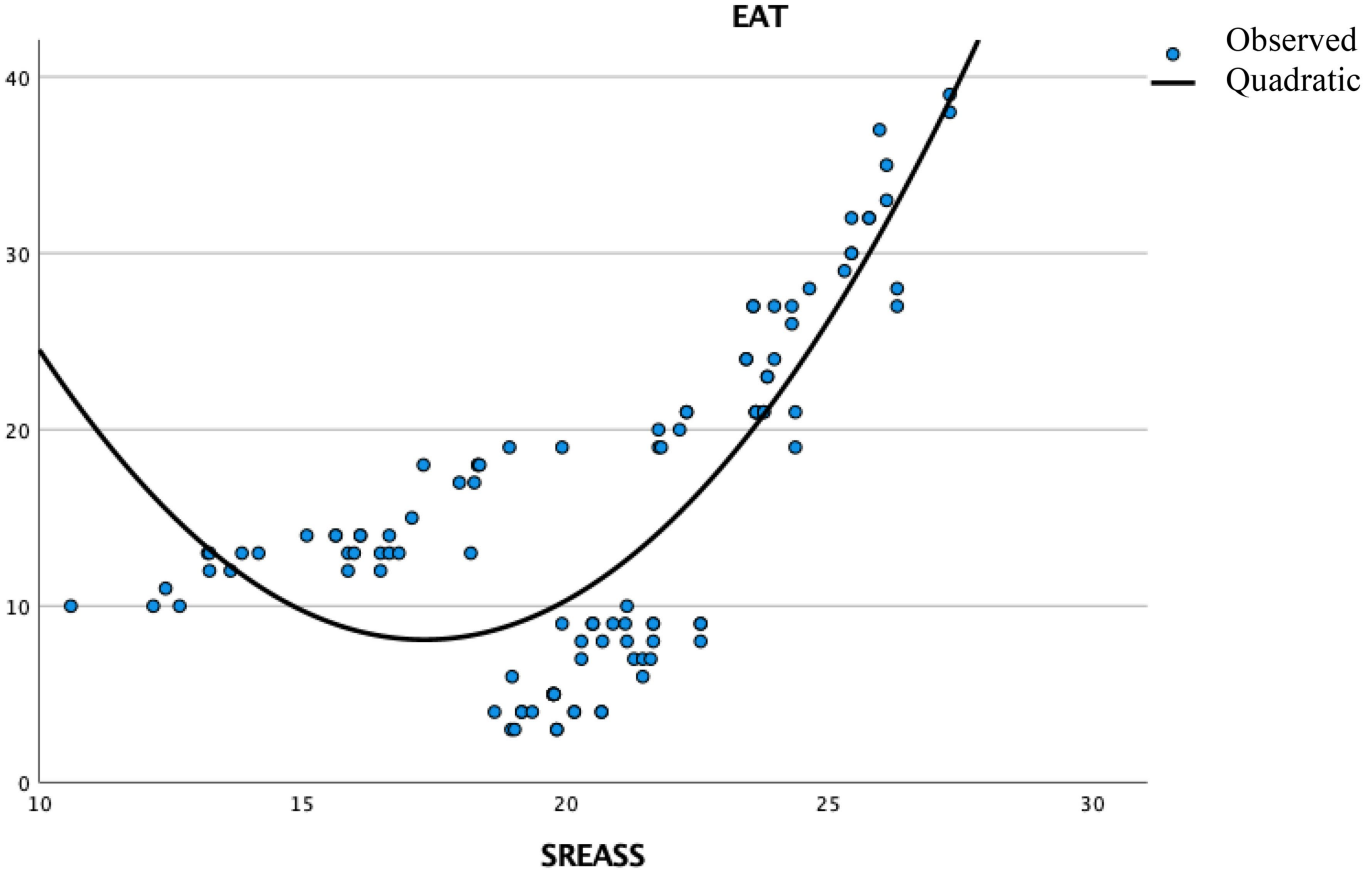
Curvilinear relations between self-regulation of eating attitudes and disordered eating.

The above results confirmed previous findings that found a linear relationship between SREASS scores, and disordered eating attitudes (Scoffier et al., 2010a). Study found a linear relationship between self-regulation of eating attitudes and disordered eating indicating a consistent association (*β* = 0.59, *p* < 0.001). Additionally, self-regulation of eating attitudes showed a significant quadratic effect on disordered eating (*β* = −4.38, *p* < 0.001). The shape of the curves reflects the too much of a good thing effect (Figure 1).

**Figure 2:**
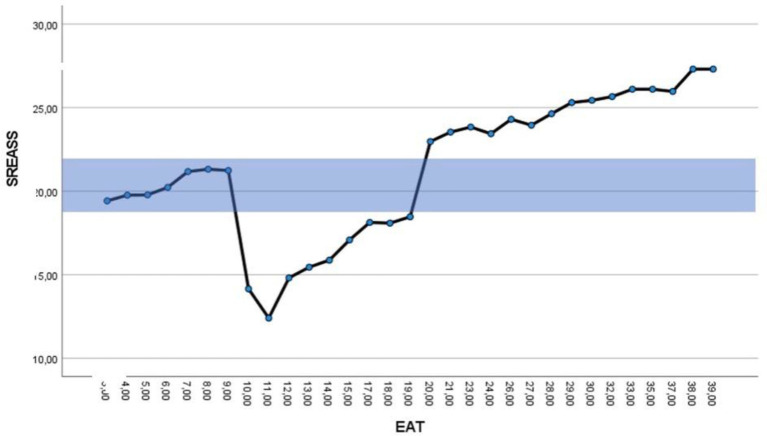
Plot of the interaction between Eating Attitudes Test (EAT) and Self-Regulatory Eating Attitude in Sports Scale (SREASS) scores. X-axis refers to EAT scores and the Y-axis refers to SREASS scores.

To better-determine the ZOREAS, we ran a one-way ANOVA which was significant for disordered eating attitudes [*F* (30; 42.16) = 21.37, *p* = 0.00]. The plot of the interaction (cf., Figure 2) confirmed three trends: (i) a high level of disordered eating attitudes (EAT scores over 20) is associated with a high level of self-regulation of eating attitudes (SREASS scores over 24); (ii) a medium level of disordered eating attitudes is associated with a low level of self-regulation of eating attitudes (SREASS scores under 19); and (iii) a low level of disordered eating attitudes is associated with a medium level of self-regulation of eating attitudes (SREASS scores between 19 and 24). Thus, the ZOREAS was determined to be SREASS scores within the range 19–24. The ZOREAS has been colored in blue on the Figure 2.

## Discussion

This study examined the relationship between the self-regulation of eating attitudes in sport with a specific tool adapted to a population of athletes and disordered eating, using instruments useful to a population of athletes considering the literature. Consistent with our hypothesis, when we consider the global sample with correlational analysis, we observed an overall, negative linear relationship between self-regulatory eating attitudes and disordered eating in the sports context. This relationship confirmed the work of AbuSabha and Achterberg (1997) and demonstrated the protective character of self-regulated eating attitudes on disordered eating in a sport context. However, this study tried to investigate deeper the relationship between self-regulation of eating attitudes and disordered eating. First, the study allows us to observe that self-regulation of eating attitude increased with disordered eating down to a point and then leveled up. So, this step reflects the too much of a good thing effect. The study also tested the hypothesis of the existence of a ZOREAS and revealed that the overall relationship between self-regulatory eating attitudes and disordered eating was not totally linear. This latter finding confirms the definition of disordered eating given in Lynch et al. (2015) or Chen et al. (2015), which considers that eating disorders relate to the over- or under-control of socioemotional behaviors. Specifically, our analyses identified a ZOREAS, characterized by SREASS scores between 19 and 24 and EAT scores very low (<10). The concept of the ZOREAS is not well-documented in the literature, and our study is the first of its kind. Based on this study, the SREASS and the ZOREAS seems to be an interesting tool for determining disordered eating risk.

### Perspectives

Future longitudinal studies could determine the stability and the specificity of the ZOREAS, while an experimental study would help to establish the direction of causality suggested by our correlational analysis. Most importantly, this type of research would help in developing educational programs based on self-regulation of eating attitude adaptation that seek to limit the emergence of disordered eating among athletes. Our study has several limitations that need to be considered. First, data were self-reported, and could be biased by social desirability. Second, correlation analyses were used, which limits the generalization of the demonstrated relationships between variables (i.e., the self-regulation of eating attitudes and disordered eating). Third, the ZOREAS could be compared to the *individual zone of optimal functioning* (Hanin, 2000), and like the latter, could vary according to the sport and the individual. Future studies could investigate the individual ZOREAS considering the eating behaviors and the performance in a longitudinal design.

### Applications for Professionals

The ZOREAS may be useful to sports psychology practitioners who work with athletes. For instance, it could provide a more holistic understanding of the degree of self-regulation of the athletes and in consequence influence how, in general, athletes manage their eating attitude in the context of their chosen sport. The ZOREAS cannot be used as a measure to determine ability to change eating strategies, but it can be used as a tool to determine if someone should change eating strategies. So, it could be used to evaluate an athlete’s ability to engage in new, and better-adapted eating strategies, and to determine whether the development of new eating attitudes would benefit performance outcomes.

## Data Availability Statement

The raw data supporting the conclusions of this article will be made available by the authors, without undue reservation.

## Ethics Statement

The studies involving human participants were reviewed and approved by the Université Côte d’Azur Ethics Committee authorization number: 2021-011. Written informed consent to participate in this study was provided by the participants’ legal guardian/next of kin.

## Author Contributions

SS-M defined the project and realized the data collection. SS-M and YP worked on the analyses and the manuscript. All authors contributed to the article and approved the submitted version.

## Conflict of Interest

The authors declare that the research was conducted in the absence of any commercial or financial relationships that could be construed as a potential conflict of interest.

## Publisher’s Note

All claims expressed in this article are solely those of the authors and do not necessarily represent those of their affiliated organizations, or those of the publisher, the editors and the reviewers. Any product that may be evaluated in this article, or claim that may be made by its manufacturer, is not guaranteed or endorsed by the publisher.

## Appendices

**Appendix 1** **|** Self-regulation of eating attitudes in sport scale (SREASS).

1. Te sens-tu capable de manger un gâteau sans penser aux conséquences que cela va pouvoir avoir pour ta prochaine compétition? *(Do you feel capable of eating a dessert without thinking of the consequences this may have on your next competition?)*

2. Te sens-tu capable d’aller te faire vomir si tu as mangé du gâteau d’anniversaire à une fête? *(Do you feel capable of making yourself vomit if you have just eaten cake at a birthday celebration?)*

3. Te sens-tu capable de contrôler ce que tu manges quand de la nourriture alléchante est. devant toi? *(Do you feel capable of controlling what you eat when tempting food is put before you?)*

4. Te sens-tu capable de contrôler ce que tu manges quand il y a beaucoup de nourriture disponible pour toi? *(Do you feel capable of controlling what you eat when a lot of food is easily available?)*

5. Te sens-tu capable de contrôler ce que tu manges quand tu es anxieux(se) ou inquiet(e)? *(Do you feel capable of controlling what you eat when you are anxious or worried?)*

6. Te sens-tu capable de contrôler ce que tu manges quand tu es irritable? *(Do you feel capable of controlling what you eat when you are irritable?)*

7. Te sens-tu capable de manger avec tes partenaires d’entraînement et ne pas te priver? *(Do you feel capable of eating with your training partners without depriving yourself?)*

8. Te sens-tu capable de manger des frites sans penser aux conséquences que cela va pouvoir avoir sur tes performances? *(Do you feel capable of eating french fries without thinking of the consequences this may have on your performance?)*

9. Te sens-tu capable de ne rien manger à un repas sous prétexte de la présence de ton entraîneur? *(Do you feel capable of eating nothing at a meal using the pretext that your coach is present?)*

10. Te sens-tu capable de contrôler ce que tu manges quand tu es déprimé(e)? *(Do you feel capable of controlling what you eat when you are depressed?)*

11. Te sens-tu capable de manger des sucreries sans penser aux conséquences que cela va pouvoir avoir sur ta prochaine compétition? *(Do you feel capable of eating sweets without thinking of the consequences this may have on your next competition?)*

12. Te sens-tu capable de manger en grosse quantité sans penser aux conséquences que cela va pouvoir avoir sur tes performances? *(Do you feel capable of a lot of food at a time without thinking of the consequence this may have of your performance?)*

13. Te sens-tu capable de manger trois repas par jour sans te faire vomir, pratiquer de l’exercice excessif, prendre des diurétiques ou des laxatifs? *(Do you feel capable of eating three meals a day without making yourself vomit, exercise to excess, or take diuretics or laxatives?)*

14. Te sens-tu capable de manger de la nourriture riche en graisses sans te faire vomir, pratique de l’exercice excessif, prendre des diurétiques ou des laxatifs? *(Do you feel capable of eating high-fat foods without making yourself vomit, exercise to excess, or take diuretics or laxatives?)*

15. Te sens-tu capable de prendre un repas avec tes parents en mangeant en quantité normale? *(Do you feel capable of eating a normal amount of food when you have a meal with your parents?)*

16. Te sens-tu capable de résister à la tentation de sucreries que tu apprécies beaucoup? *(Do you feel capable of resisting the sweet foods that you like the most?)*

Inversed items: 2 and 9.

**Appendix 2** **|** Eating attitudes test 26 (Garner et al., 1982).

1. I am terrified about being overweight.

2. I avoid eating when I am hungry.

3. I find myself preoccupied with food.

4. I have gone on eating binges where I feel that I may not be able to stop.

5. I cut my food into small pieces.

6. I aware of the calorie content of foods that I eat.

7. I particularly avoid food with a high carbohydrate content (i.e., bread, rice, potatoes, etc.).

8. I feel that others would prefer if I ate more.

9. I vomit after I have eaten.

10. I feel extremely guilty after eating.

11. I am occupied with a desire to be thinner.

12. I think about burning up calories when I exercise.

13. I other people think that I am too thin.

14. I am preoccupied with the thought of having fat on my body.

15. I take longer than others to eat my meals.

16. I avoid foods with sugar in them.

17. I eat diet foods.

18. I feel that food controls my life.

19. I display self-control around food.

20. I feel that others pressure me to eat.

21. I give too much time and thought to food.

22. I feel uncomfortable after eating sweets.

23. I engage in dieting behavior.

24. I like my stomach to be empty.

25. I have the impulse to vomit after meals.

26. I enjoy trying new rich foods.

Inversed item: 26.
